# Surgical Management of Brain Metastasis: Challenges and Nuances

**DOI:** 10.3389/fonc.2022.847110

**Published:** 2022-03-14

**Authors:** Chibawanye I. Ene, Sherise D. Ferguson

**Affiliations:** Department of Neurosurgery, University of Texas, MD Anderson Cancer Center, Houston, TX, United States

**Keywords:** brain metastasis, surgery, en bloc resection, LITT, LMD

## Abstract

Brain metastasis is the most common type of intracranial tumor. The contemporary management of brain metastasis is a challenging issue and traditionally has carried a poor prognosis as these lesions typically occur in the setting of advanced cancer. However, improvement in systemic therapy, advances in radiation techniques and multimodal therapy tailored to the individual patient, has given hope to this patient population. Surgical resection has a well-established role in the management of brain metastasis. Here we discuss the evolving role of surgery in the treatment of this diverse patient population.

## Introduction

Brain metastases represent the most common brain tumors in adults in the United States and outnumber primary brain tumors 5:1 (1, 2). Approximately 8-10% of patients with systemic cancer will develop brain metastasis (3–5). Lung cancer, breast cancer and melanoma represent the most common solid tumor pathologies to develop brain metastasis. Melanoma has the highest frequency with 40-60% of patients developing brain metastasis.6 While 37-50% of patients present with single brain metastasis, 50-63% have multiple brain lesions at presentation (6, 7). Historically brain metastasis prognosis is quite poor and more than half of the patients diagnosed with brain metastasis will die within 3-27 months of diagnosis (3–5). With advances in systemic therapy patients are living longer with advanced cancer with more opportunity to develop brain metastases (5–9). Brain metastasis represent a major source of morbidity in cancer patients and are a source of significant social and economic burden for patients and caregivers (10). Management of patients with brain metastases is complex and best performed by multispecialty teams consisting of medical oncologists, surgeons, and radiation oncologists and team members must appreciate the nuances of the available treatment paradigms in order to tailor individualized care. Surgery remains the cornerstone in brain metastasis management. Here we outline the surgical management of brain metastasis focusing on surgical challenges, nuances and decision-making.

## Surgical Management of Single/Solitary Brain Metastasis

A solitary brain metastasis is defined as one brain lesion without evidence of extracranial metastasis, whereas a single brain metastasis is one brain lesion with at least one other site of extracranial disease. The essential role of surgery in the treatment of single/solitary brain metastases is firmly established. Specifically, surgery can provide multiple pragmatic clinical benefits particularly in the setting of a large (i.e. >2.5 cm maximal diameter) symptomatic lesion. Surgical resection is the most effective way to rapidly relieve mass effect, achieve cerebral decompression and subsequently reduce intracranial pressure (ICP). Further, brain metastases often cause cerebral edema, which can be severe and contribute to worsening neurological status. Steroid administration is typically the first option to address edema, but in the circumstance of refractory symptomatic edema, tumor resection is beneficial. Resection also reduces the length of time patients require steroid treatment thereby potentially limiting the development of steroid-induced medical complications. Lesions located in the posterior fossa (e.g. cerebellum) or intraventricular metastases can obstruct cerebrospinal fluid (CSF) flow resulting in hydrocephalus, which can also be addressed with resection of the obstructing mass. Additionally, brain metastases can cause seizures due to irritation of the surrounding cortex and surgery may help in optimizing seizure control. Finally, surgery can aid diagnosis when the etiology/pathology of the brain lesion is unclear; specifically in the circumstance of a new brain lesion with a negative systemic workup or in a patient with a history of an unknown primary. Notably approximately 11% of patients with a diagnosis of a primary cancer may have a non-metastatic brain lesion such as glioma (11). As such, if imaging characteristics favor a primary glial neoplasm, surgical biopsy may be warranted to guide the subsequent treatment plan.

In addition to the clinical benefits, surgical resection also provides a known survival advantage in the setting of single metastasis. The positive impact of surgery was solidified after the completion of two pivotal randomized clinical trials. The first was conducted by Patchell and colleagues, who randomized patients with a single brain metastasis to receive tumor resection followed by whole-brain radiation therapy (WBRT) (n = 25) versus WBRT alone (n = 23) (11). The authors found that patients in the surgical resection group survived significantly longer than patients treated with WBRT alone (median survival of 40 weeks versus 15 weeks, respectively). Surgery was also associated with significantly lower risk of local recurrence (20%) relative to WBRT alone (52%). Finally, surgical patients maintained functional independence [defined by a Karnofsky Performance Scale (KPS) score of >70] significantly longer (median, 38 weeks) relative to patients treated with only WBRT (median, 8 weeks). A second prospective randomized study by Vecht et al. also compared combination surgical resection plus radiation versus radiation alone in patients with a single brain lesion (12). Primary outcomes measures were overall survival and functionally independent survival (FIS). Combined treatment led to longer patient survival (*p =* 0.04) and a longer FIS (*p =* 0.06) compared with radiotherapy alone. This was most pronounced in patients with stable extracranial disease (median survival, 12 versus 7 months; median FIS, 9 versus 4 months). Overall, these two historic trials verified the substantial advantage surgery imparts.

In the modern treatment of single/solitary brain metastases, WBRT has given way to stereotactic radiosurgery (SRS) as an upfront treatment option, primarily due to the detrimental cognitive effects of WBRT (13–15). SRS is a specialized radiation technique in which a targeted dose of radiation is delivered to one or more intracranial lesions with high precision. SRS can be delivered in a single or multiple fractions and has become a standard of care in the management of brain metastasis. Even with the availability of this effective, minimally invasive treatment option, surgery continues to play a powerful role, particularly in the setting of large brain metastases. Prabhu et al. conducted a retrospective analysis of 213 patients with large brain metastases treated with single fraction SRS alone or surgery + SRS between 2005 and 2013 from two institutions (16). In this study, large brain metastases were defined as ≥4 cm^3^ (2 cm in diameter) and surgical gross total resection (GTR) was required for inclusion. Overall, 213 patients with 223 treated brain metastases were included; 66 (30%) were treated with SRS alone and 157 (70%) with combination surgery + SRS (pre-operative or post-operative). Patients in the combination therapy group had higher tumor volumes (median 9.6 cm^3^) compared to patients receiving SRS alone (5.9 cm^3^; p<0.001). Patients receiving surgery + SRS demonstrated significantly longer survival compared with those receiving SRS alone with a median survival of 15.2 months versus 10 months (*p* < 0.01) respectively. Overall survival was significantly higher in the surgery + SRS group (2-year OS rate, 38.9% vs 19.8%; *p*= 0.01). Finally, the local recurrence (LR) rate was significantly lower with surgery + SRS (1-year LR rate, 36.7% versus 20.5%; *p*= 0.07) (16). This study highlights the critical role of surgery even with the availability of SRS.

### Patient Selection

Thoughtful patient selection is the foundation of surgical decision-making. The survival benefit of surgical resection can be significantly diluted if surgical candidates are not carefully selected. Brain metastasis patients are a challenging population with unique factors that should be balanced when considering surgery. As brain metastases are often a consequence of advanced systemic cancer, many patients are elderly and may have age-related medical co-morbidities that increase surgical risk (17). Cancer patients are higher risk for thromboembolic complications (e.g. deep vein thrombosis, pulmonary emboli) throughout the course of their illness requiring anti-coagulation and this must be taken into consideration for surgical planning to reduce the risk of intra- and post-operative bleeding complications. Furthermore, venous thromboembolic complications are reported to be the most common post-operative medical complication of brain metastases surgery (17). Another critical consideration is that surgery will typically delay the initiation of systemic therapy and/or radiation for weeks to allow for post-operative healing. Notably, even minor post-operative wound healing issues or surgical site infections can delay therapy even longer and be detrimental to patient care, particularly if re-operation/open surgical debridement is required to address infection. Finally, metastases in eloquent cortex (motor and language centers) pose a particular concern as the development of a new neurological deficit can significantly impact quality of life. Further, a major post-operative neurological deficit may significantly reduce a patient’s functional status and harm candidacy for aggressive adjuvant therapy and/or clinical trial enrollment. Additionally, surgeries in functional cortical locations may require longer recovery and rehabilitation times, which must be carefully balanced with a patient’s life expectancy.

Overall, younger patients (<65 years) with high functional status (KPS score ≥ 70), controlled systemic disease and no extracranial metastases are considered to be the most suitable candidates based on the classic recursive partitioning analysis (RPA) classification system developed by the RTOG (Radiation Therapy Oncology Group). In a pivotal study by Tenduklar et al. (18) the authors analyzed the outcome of 271 patients undergoing resection for a solitary brain metastasis. They reported that patient survival was significantly correlated with RPA class and specifically patients with the above-mentioned attributes had the best prognosis suggesting this patient population most suitable for surgical resection (18). A diagnosis specific graded prognostic assessment (GPA) is another prognostic algorithm that accounts for tumor histology and was developed based on the analysis of over 4000 patients with brain metastasis including breast, lung, GI, melanoma and renal cell carcinoma (19–21).

Even though the characteristics of the “ideal” surgical candidate are well described, there are circumstances where surgery may be considered in patients that do not meet these specific criteria. First in the setting of an emergency, there is likely not adequate time for establishment of systemic disease status prior to proceeding with resection as the priority would be to immediately relieve life-threatening elevated ICP. Second, patient functional status at presentation may be modifiable and improved with surgical intervention. For example, a patient may present with a metastasis in the motor area causing hemiplegia and an associated low functional status. However, resection of the symptomatic lesion can restore functional status, improve KPS and allow the patient to be a candidate for systemic therapy post-operatively. Hence, surgery may be considered in a patient with lower functional status, if that condition is a direct consequence of the metastatic lesion and is potentially reversible. Third, in regards to patients with uncontrolled systemic disease, it may be prudent to consider whether the patient’s uncontrolled disease is at initial presentation (where systemic treatment options remain available) or in the setting of refractory disease progression and multiple failed treatment regimens as these represent vastly different clinical scenarios. Moreover, with advances in molecular testing, targeted therapy and immunotherapy, subpopulations with advanced disease are surviving longer so surgical consideration may be at times reasonable, particularly if the patient is symptomatic. Additionally, it is important to note that the classic prognostic algorithms do not factor in patient medical co-morbidities that can impact post-operative morbidity, re-admission and mortality (17). The current algorithms focus on age, however a healthy, 75-year old patient with no medical comorbidities maybe be a more desirable surgical candidate than a 60 year-old with multiple crippling medial ailments. Overall, the careful consideration of surgical candidacy is critical in the management of brain metastasis. Brain metastasis patients represent a complex population patient, selection for surgical resection can be highly nuanced and a multi-disciplinary evaluation is invaluable.

### Impact of Surgical Technique

The maximal benefit of surgical resection is dependent on both extent of resection and surgical technique. In metastasis surgery, radiographic gross total resection (GTR) is the goal whenever feasible as it improves patient outcome (18, 22). A retrospective analysis of 271 patients from single institution (1984-2004) found that GTR of metastasis was associated with a median overall survival of 10.6 months versus subtotal resection (STR; 8.7 months) (18). Although these results did not reach statistical significance (*p*=0.07). A more recent study (1995-2011) retrospectively evaluated the outcomes of 157 patients with single brain metastasis and reported a median post-operative survival of 19.3 months (22). Among the 157 patients; the majority received post-operative radiation; whole-brain radiotherapy (11%) and radiosurgery (69%). Multivariate analysis showed that extent of surgical resection was significantly correlated with survival. Median survival was 20.4 months following GTR and 15.1 months after STR (*p*= 0.016).

In addition to extent of resection, there is substantial data underscoring the importance of surgical technique on the outcome of brain metastasis surgery. Historically, brain metastasis surgery was often accomplished *via* a piecemeal resection. This method of resection involves internal debulking of the mass followed by removal of the tumor capsule in multiple pieces. En bloc resection, on the other hand, entails circumferential dissection of the tumor along the brain-tumor interface without violating the tumor capsule. This method avoids spillage of tumor contents into the resection cavity. From a purely technical standpoint, en bloc resection is helpful as brain metastasis can be vascular and dissection along the tumor margin (as opposed to entering a vascular lesion) may reduce intraoperative bleeding and reduce operative time. Furthermore, dissection along the brain-tumor interface allows for better definition of tumor borders, aiding in the accomplishment of a complete resection.

Beyond the technical aspects, en bloc resection also positively impacts patient outcome. In a landmark study by Patel et al., the authors evaluated the predictors of local recurrence after resection of untreated single brain metastasis. This was a single institution study that included 570 surgical cases; 35% of cases done with a piecemeal resection technique and 65% en bloc. The overall rate of local recurrence was 15%. This study identified two factors that impacted local recurrence: tumor volume (greater than 9.7cm^3^) and resection technique. Specifically, the authors reported that patients who had piecemeal GTR were 1.7 times more likely to develop local recurrence compared to patients who had an en bloc resection (*p* = 0.03) (23). This was one of the earliest studies advocating for en bloc resection. A follow-up study at the same institution, which included an analysis of 1033 patients with single brain metastases also determined that en bloc resection was not associated with increased complication rates even for tumors in functional areas of the brain (i.e. eloquent cortex) emphasizing that en bloc resection is both effective and safe (24).

In addition to local recurrence, distance recurrence and/or the development of leptomeningeal spread is major concern in the management of brain metastases. Leptomeningeal disease (LMD), which entails tumor spread to the leptomeninges and/or CSF, is a devastating form of metastatic dissemination associated with a very poor prognosis (25, 26). Notably, another reported advantage of en bloc resection includes a lower risk of LMD. A single institution study examined the risk of LMD following resection of posterior fossa metastasis (27). Posterior fossa/infratentorial (e.g. cerebellum/vermis) metastases are of particular concern for LMD due to their proximity to ventricular/CSF spaces and the opportunity for CSF spread. In this study, Suki et al., analyzed the outcome of 379 patients with posterior lesions undergoing either SRS (n = 119) or open surgical resection (n = 260). The primary outcome measure was development of LMD. Interestingly piecemeal resection was associated with significantly higher LMD risk compared to en bloc resection (*p* = 0.006) or SRS (*p* = 0.006). Specifically, of the patients undergoing en bloc resection only 5.7% developed LMD compared with 13.9% of piecemeal resection patients. It is hypothesized that an en bloc resection provides this advantage because it avoids violation of the tumor capsule, which could lead to spillage of tumor contents into CSF space. A similar investigation was conducted in patients with supratentorial brain metastasis (28). This study included 827 patients with a supratentorial brain metastasis that underwent surgical resection (191 piecemeal and 351 en bloc) or SRS (n = 295). Once again the authors reported that en bloc resection was associated with a lower incidence of LMD compared to piecemeal resection. This difference was most pronounced in patients with melanoma brain metastases (28).

## Surgical Management of Multiple Brain Metatases

Approximately 30–50% of brain metastasis patients present with multiple lesions (6, 7). In contrast to single/solitary brain metastasis, in which the beneficial role of surgical resection has been established by prospective, randomized trials (11, 12), no class I evidence exists for the role of surgery patients with multiple brain metastases. There are specifically are no prospective randomized studies formally evaluating the impact of surgery on patient survival in the setting of multiple brain metastases. However, in patients with multiple brain metastases, surgery may be beneficial to provide symptomatic relief and/or improve KPS, particularly after resection of large dominant lesion(s). In a recent multi-center, retrospective study, the authors analyzed the outcome of 750 surgical patients following resection (29). This study included patients with multiple brain metastases (39% of cases). The authors reported that functional status was significantly improved by surgical resection, with a median preoperative KPS of 80 increasing to 90 post-resection (*p*<0.0001). Furthermore, systemic treatment was more frequently provided to patients with KPS >70 (*p*<0.0001) and this was associated with improved patient survival (16 versus 7 months; *p*<0.0001).

Even though prospective data is limited, retrospective studies on multiple brain metastases indicate that the best survival outcome is obtained when all lesions are resected if feasible (30–32). Bindal et al. evaluated 56 patients who underwent resection for multiple brain metastases (30). Thirty patients had one or more lesions left unresected (Group A) and 26 patients had all lesions resected (Group B). This study also included a matched cohort of patients with a single metastasis resected for comparison (n = 26; Group C). These authors reported that symptoms improved in 65% of Group A patients compared to 83% in Group B. Furthermore, the survival of patients who had all lesions resected was also significantly longer than in patients who had residual lesions (14 versus 6 months respectively). Notably there was no significant survival difference between patients who had multiple metastases with complete resection of all (Group B) and those who had a single metastasis removed (Group C) (30). These results have been duplicated in other surgical series. Salvalti et al. retrospectively analyzed the outcome of 32 patients undergoing resection for multiple brain metastases (2-3 lesions). They compared the outcome of this cohort to 30 patients undergoing resection for a single brain metastasis. Neurological status improved in approximately 60% of multiple metastases patients post resection and there was no significant difference in survival between patients who had multiple metastases resected compared to those with a single lesion resected (31). Another study by Schakert et al. evaluated 127 patients with multiple brain metastasis (32). Similar to the prior study, patients who had all lesions resected had prolonged survival compared to patients with residual lesions (10.6 versus 5.8 months respectively) (32).

In summary, there is increasing data to support surgical resection in patients with multiple brain metastases. Resection can improve functional outcome and potentially improve candidacy for adjuvant therapy, which is critical to overall cancer prognosis. However, most studies are small series and larger prospective studies are needed. Additionally, the majority of surgical series are not pathology specific. With advances in molecular profiling and targeted systemic treatments, tumor specific studies are warranted to fully capture the benefit of resection in this population.

## Surgical Management of Recurrent Brain Metatases and Radiation Necrosis

Even with maximal therapy, including resection, brain metastases can recur locally or distantly, requiring further intervention. The challenge is that most patients with recurrent lesions have already undergone extensive cranial treatment (resection, SRS, and/or WBRT), limiting additional therapeutic options. In the setting of large, symptomatic, and/or previously treated brain metastases, repeat surgical resection is a reasonable treatment option in appropriately selected patients. A retrospective analysis reported the outcome of 67 patients with recurrent brain metastasis undergoing repeat resection. All patients had surgery as a component of their initial treatment. The majority of patients had a distant recurrence (n = 35) and GTR was achieved in most patients with solitary metastases. The overall median post-operative survival time was 7.5 months. Multivariate analysis demonstrated that RPA class and time to recurrence were both significant predictors of patient survival. Specifically, in patients who recurred occurred within 200 days of the initial resection, the median survival time was only 6 months compared with patients who recurred after 200 days (9.2 months) (33).

Management of brain metastasis after failed SRS is a particular challenge. Local progression and/or radiation necrosis is reported to occur in approximately 20% of brain metastasis treated with SRS (34–36). This patient population will pose a growing concern as it becomes more common to treat a higher number of brain metastases with upfront SRS in order to avoid the cognitive side effects of WBRT (13, 37). Each lesion treated with SRS theoretically has the potential to fail or develop into radiation necrosis. Radiation necrosis is a known complication of SRS and is characterized by a progressive radiation-induced inflammatory reaction, which can result in neurological symptoms (38, 39). It can be a difficult condition to manage for several reasons. First, radiographically, it can be difficult to distinguish radiation necrosis from true tumor progression as both enhance on post contrast imaging and can cause cerebral edema and mass effect. Even with advanced imaging modalities such as MR mass spectroscopy, perfusion and diffusion studies and positron emission tomography (PET) (40–46), diagnosis cannot be confirmed without pathological diagnosis. The correct diagnosis can be critical for deciding the next treatment step since radiation necrosis can be observed (especially if small and/or asymptomatic) while tumor progression necessitates treatment escalation. Furthermore, the results of imaging studies can be inconclusive and these recurrent/failed treatment lesions can sometimes be mixed, with components of both radiation necrosis and active progressive tumor. Second, patients with radiation necrosis can have severe symptoms; particularly because the intense inflammatory reaction can cause extensive cerebral edema, which sometimes can be disproportionate to the size of the enhancing mass itself. Third, often the first line of therapy is steroid treatment to reduce symptomatic edema. And for some patients, a slow steroid taper (over 2-3 weeks) will be sufficient to address clinical symptoms and stabilize or improve radiographic changes without the need for additional intervention. However, in a subset of patients, radiation necrosis can become progressive and refractory to steroid therapy. Since long-term steroid use is suboptimal due to risk of medical complications, in such cases additional interventions may be required. There are effective medical therapies such as Avastin (bevacizumab), which has shown notable benefit in the treatment of radiation necrosis (47, 48) however; we will focus the surgical treatment options.

There are several studies evaluating the effectiveness of salvage surgery for recurrent metastasis/failed SRS/radiation necrosis (49–53). Kano et al., retrospectively analyzed the outcome of 58 patients who required resection of brain metastases following previous treatment with SRS. Median time between SRS and surgical resection was 7.1 months. Median follow-up was 7.6 months and median overall survival following resection was 7.7 months. Post-operatively, the local tumor control rate was 62% at 12 months and peri-operative morbidity was reported in 6.9% of cases. Interestingly these authors reported that a short interval between initial SRS and surgical resection (< 3 months) was significantly associated with poor survival (*p* = 0.001). In fact, no patient having a salvage surgery within three months of SRS lived more than one year post-operatively. This finding is an important consideration in determining surgical candidacy in the setting of recurrent metastasis (49). A more recent study by Mitsuya et al. (50), also evaluated the efficacy of salvage surgery in a cohort of 48 surgical patients (54 surgeries). In this study the median post-operative survival was 20 months with a reported local control rate of 76% at one year. Further, this study highlighted the palliative benefit of surgery as among the patients with pre-operative neurological deficits, 75% of cases had neurological improvement following salvage surgical resection (50). Overall, in select patients, salvage surgery for failed SRS is a reasonable treatment option that can aid in symptom management with a reasonable rate of local control and low surgical mortality.

In cases were surgical resection is not feasible due to an inaccessible location or suboptimal patient candidacy for an open craniotomy, laser interstitial thermal therapy (LITT) may serve as a salvage treatment option (54–57). The principle of LITT is selective ablation of target tissue by heat. Laser electromagnetic radiation is focused energy that is transformed into thermal energy, which spreads to tissues to induce coagulation. LITT is a minimally invasive procedure that consists of a probe inserted under stereotactic guidance into the target lesion. When the laser interacts with the target tissue, the tissue absorbs the laser photons, which are then transformed into thermal energy inside the target tissue. The heat generated heat leads to thermal damage of the target tissue with the goal of inducing necrosis through protein denaturation, while avoiding damage to surrounding normal tissues (57, 58). The ideal lesion for LITT is a well-circumscribed lesion with a diameter 3-3.5 cm or less (57). For larger lesions; multiple fibers could be used to cover the entire target. Lesions located inside the ventricles or near heat sink areas (porencephalic cysts, dura large venous lakes, large caliber vessels) might represent a challenge for thermal spread and should be evaluated on a case-by-case basis (57).

The main advantage of LITT is the ability to treat lesions not amenable to surgical resection due to difficult locations. The minimally invasive nature of the procedures allows for potentially shorter hospital stays, and faster transition to adjuvant treatments. LITT can also be repeated if progression is found after the procedure with no concern of accumulated ionizing radiation damage. LITT also it does not preclude a future open surgery in the case of treatment failure. Bastos et al. performed a retrospective study with consecutive brain metastasis patients treated with LITT (59). Based on radiological aspects, lesions were divided into progressive disease after SRS (recurrence or radiation necrosis) and new, untreated lesions. The primary endpoint was time to local recurrence. A total of 61 consecutive patients with 82 lesions (5 newly diagnosed, 46 recurrence, and 31 radiation necrosis) were included for analysis. Freedom from local recurrence at 6 months was 69.6% and 59.4% at 12, months. Shorter time to recurrence was significantly associated with incompletely ablated lesions (*p* <.001), larger lesion volume (>6 cc) (*p* = 0.03) and dural-based lesions (*p* = 0.01). Tumor recurrence/newly diagnosed metastases also had shorter time to local recurrence when compared to radiation necrosis (*p* = 0.01). Patients receiving systemic therapy after LITT had longer time to local recurrence (*p* = 0.01). In multivariate analysis the hazard ratio for incompletely ablated lesions was 4.88 (*p* <.001), 3.12 (*p*= 0.03) for recurrent tumors, and 2.56 (*p* = 0.02) for patients not receiving systemic therapy after LITT. The procedural complication rate in this series was 26%. Notably, this complication rate is higher than reported in surgical resection series. However, it is important to consider that patients may be dispositioned for LITT due to lesions in high-risk locations and/or higher risk medical conditions, potentially contributing the elevated complication rate. One additional consideration is the initial inflammatory response caused by LITT, which typically requires steroids post-operatively. This period can be prolonged depending on the duration of previous steroid use and degree of pre-operative peri-lesional edema. However, a recent study comparing the post-operative outcome of LITT versus craniotomy for failed SRS lesions reported no significant differences in the rates of steroid cessation at 1-month follow-up between the LITT and surgical resection (60). The authors also reported no significant delay in the resumption or initiation of immunotherapy between the two treatment modalities, which is an important consideration in the setting of extended steroid use. In summary, LITT is a valid salvage strategy for recurrent brain metastasis or radiation necrosis. The current data would be strengthened by a prospective, randomized study.

## Conclusion

As patients live longer with advanced cancer, brain metastasis will continue to be a growing issue. Brain metastases are a major contributor to cancer mortality and can have a significant impact on patient quality of life. Even with the significant advances in systemic therapy and radiation techniques, surgery remains a critical aspect of patient management. The maximal benefit of surgery can be achieved with careful patient selection and attention to surgical technique.

## Author Contributions

SF and CE: Conception, drafting of manuscript and review of final version. All authors contributed to the article and approved the submitted version.

## Conflict of Interest

The authors declare that the research was conducted in the absence of any commercial or financial relationships that could be construed as a potential conflict of interest.

## Publisher’s Note

All claims expressed in this article are solely those of the authors and do not necessarily represent those of their affiliated organizations, or those of the publisher, the editors and the reviewers. Any product that may be evaluated in this article, or claim that may be made by its manufacturer, is not guaranteed or endorsed by the publisher.
